# A network meta-analysis: the overall and progression-free survival of glioma patients treated by different chemotherapeutic interventions combined with radiation therapy (RT)

**DOI:** 10.18632/oncotarget.10763

**Published:** 2016-07-21

**Authors:** Ling Qi, Lijuan Ding, Shuran Wang, Yue Zhong, Donghai Zhao, Ling Gao, Weiyao Wang, Peng Lv, Ye Xu, Shudong Wang

**Affiliations:** ^1^ Basic Medical College, Jilin Medical University, Jilin, China; ^2^ Department of Radiation Oncology, First Hospital of Jilin University, Changchun, China; ^3^ Department of Science and Technology, Jilin Medical University, Jilin, China; ^4^ Center of Cardiovascular Diseases, First Hospital of Jilin University, Changchun, China

**Keywords:** chemotherapy, radiotherapy, glioma, network meta-analysis, temozolomide

## Abstract

Different chemotherapy drugs are generally introduced in clinical practices combining with therapy for glioma treatment. However, these chemotherapy drugs have rarely been compared with each other and the optimum drug still remains to be proved. In this research, medical databases were consulted, PubMed, Embase and Cochrane Library included. As primary outcomes, hazard ratio (HR) of overall survival (OS) and progression-free survival (PFS) with their corresponding 95% credential intervals (*CrI*) were reported. A network meta-analysis was conducted; the surface under the cumulative ranking curve (SUCRA) was utilized for treatment rank and a cluster analysis based on SUCRA values was performed. This research includes 14 trials with 3,681 subjects and eight interventions. In terms of network meta-analysis, placebo was proved to be inferior to the combination of temozolomide (TMZ), nimustine (ACNU) and cisplatin (CDDP). Also, bevacizumab (BEV) in conjunction with TMZ were significantly more effective than placebo with an HR of 0.40. The estimated probabilities from SUCRA verified the above outcomes, confirming that the combination of TMZ, ACNU and CDDP exhibited the highest ranking probability of 0.889 with respect to OS, while BEV in combination with TMZ - with a probability of 0.772 - ranked the first place with respect to PFS. According to the results of this network meta-analysis, the combination of (1) TMZ, ACNU and CDDP; (2) BEV in combination with TMZ and (3) cilengitide in combination with TMZ, are considered as the preferable choices of chemotherapy drugs for glioma treatment.

## INTRODUCTION

Glioma tumours developed from neoplastic glial cells - which provide support and protection for the peripheral and central nerve system [1] are a common type of primary brain tumours. This specific type of tumour accounts for approximately 30% of all brain and spine tumours and 80% of all malignant brain tumours [2]. As suggested by World Health Organization (WHO), gliomas can be further classified into four stages on the basis of their histopathological patterns, the presence of nuclear pleomorphism, the degree of increased mitotic activity and cellularity, endothelial cell proliferation and the degree of necrosis [4]. In stage I and II, gliomas are considered as low-risk cases in which more optimistic prognosis are usually observed whereas gliomas in stage III and IV are classified as high-risk cases and referred to as malignant tumours. For instance, anaplastic astrocytomas (AA) and glioblastomas (GB) are common types of stage III and IV glioma cases, and the five-year survival rate of GB patients is less than 3% [5]. Another key factor for the prognosis of glioma patients is the onset age and a large number of studies have concluded that the average survival time of GB patients was negatively correlated to their ages at which GB was diagnosed [6].

Surgery, radiation therapy (RT) or chemotherapy is introduced when oncology is certified. Surgical approaches usually involve biopsy and resection for which the extent and timing are essential factors for prognosis. Numerous studies have indicated that the extensive or gross total surgery is correlated with a less recurrent incidence and longer survival time compared with the approach of limited surgery [7-9].. RT is usually conducted for HGG patients once surgeries had been introduced to these patients and it is also appropriate for some patients who are not eligible for surgeries. Local RT, three-dimensional conformal RT and stereotactic radiosurgery are three popular RT techniques introduced in clinical practices. Apart from that, brachytherapy, radiosurgery and hyper fractionation have been introduced in order to improve remedial outcomes [11]. For instance, temozolomide (TMZ), a DNA alkylating agent, has been introduced into RT and this approach doubled the two-year survival rate to approximately 27% compared to single RT [12].

Furthermore, anti-tumour alkylating agents including carmustine (BCNU), nimustine (ACNU), lomustine (CCNU) and other nitrosourea agents also play important roles in glioma treatment. For recurrent GB, biological agents and monoclonal antibodies such as bevacizumab (BEV) are highly recommended in clinical practices [12]. Since single chemotherapy agent was limited in its effectiveness for suppressing tumour cells, two or more chemotherapy agents with synergistic effects have been introduced. Procarbazine, lomustine as well as vincristine (PCV) can be considered as commom choices. Although RT in conjunction with chemotherapy has enhanced the effectiveness of treatment, it is still challenging to determine the optimal combination of treatments due to the wide range of available interventions.

There are a large amount of studies comparing the prognosis of patients who had been treated with mono or combined anti-glioma drugs. However, the majority of evidence was generated by pair-wise meta-analyses in which only head-to-head trails were compared. Some results appeared to be biased due to the lack of study subjects. Others appeared to have contradictory results which were misleading in the current literature. This search enabled us to compared the overall survival (OS) and progression-freesurvival (PFS) status of glioma patients who had been treated with: (1) TMZ, (2) PCV, (3) BEV combined with TMZ, (4) Nimotuzumab, (5) Cilengitide combined with TMZ, (6) TMZ combined with ACNU and cisplatin (CDDP), (7) dibromodulcitol (DBD) combined with BCNU and (8) alpha-difluoromethylornithine (DMFO) combined with PCV.

## RESULTS

### Included studies

**Figure 1:**
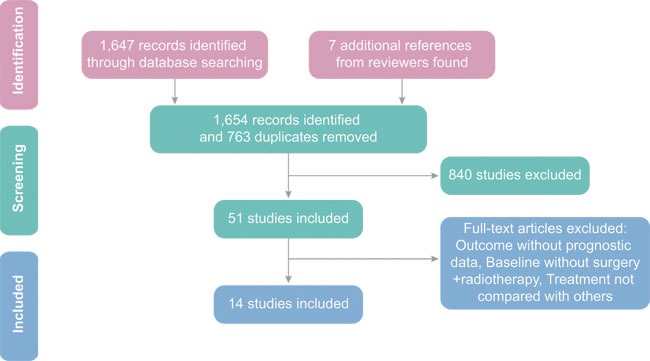
Giloma flow diagram on sampling of meta-analysis.

Fourteen eligible trials including 3,681 subjects were selected from 51 studies assessed in this meta-analysis [19-32]. Initially, 1,647 publications were identified by keywords searching in three mentioned medical databases and 41 of them are meta-analysis. Another 69 meta-analyses were found and included through reviewing references of relevant literature manually. Based on the content of abstracts and the full text of scientific papers, 51 studies were selected with 37 of the 51 studies excluded since interventions in these studies cannot form a closed loop. This resulted in the inclusion of 14 scientific papers that were published between 2000 and 2015. The entire process of literature search is illustrated in Figure 1. These 14 papers mentioned eight distinctive chemotherapy drugs that can be used for glioma: (1) TMZ, (2) PCV, (3) BEV in combination with TMZ, (4) Nimotuzumab, (5) Cilengitide in combination with TMZ, (6) TMZ combined with ACNU and CDDP, (7) DBD in combination with BCNU, and (8) DMFO in combination with PCV. Jadad scale of 14 included studies is summarized in Table S1.

### Characteristics of included trials

**Figure 2:**
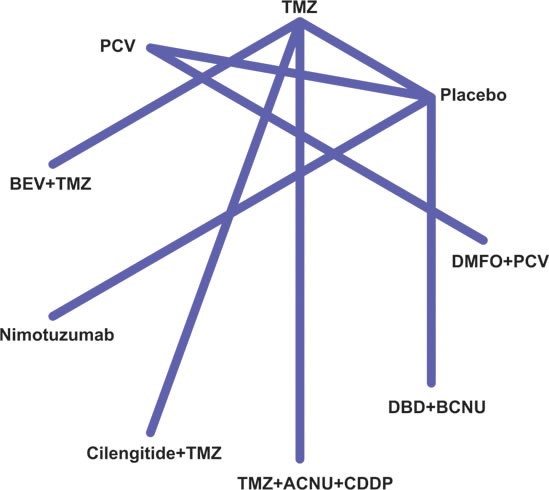
Network of treatment strategies for glioma patients of included studies.

The main characteristics and primary outcomes of the included studies and subjects are summarized in Table 1. The majority of selected studies focused on HGG patients who were either classified as GB, GBM or anaplastic oligodendroglioma/oligoastrocytomas (AA/AOA). There were seven studies which included a total of 2,405 patients with GB, while only one study investigated GBM with a total of 272 subjects. On the other hand, a randomized clinical trial with a sample of 251 LGG patients was included in our research. Besides that, only half of the 14 studies disclosed that whether glioma were newly formed cases whereas the other half of the studies failed to report this issue. All included patients have undergone radio therapeutic treatment and surgery. The entire network of comparisons among all eligible studies is demonstrated in Figure 2.

**Table 1:** Main characteristics of included studies

Author	Year	Country	Disease	Situation	Surgery	Radiotherapy	Experimental Group		Control Group		OS (HR and 95%CI)	PFS (HR and 95%CI)
							Size	Drugs	Size	Drugs*		
Solomon	2013	Cuba	HGG	-^#^	Biopsy/Resection	√	38	E	32	A	0.68 (0.42,1.11)	0.75 (0.49,1.16)
Stupp	2005	Switzerland	GB	Newly	Biopsy/Resection	√	287	B	286	A	0.63 (0.52,0.75)	0.54 (0.45,0.64)
van den Bent	2006	Netherland	AO/AOA	-	Biopsy/Resection	√	185	C	183	A	0.85 (0.65,1.11)	0.68 (0.53,0.87)
Shaw	2012	USA	LGG	-	Biopsy/Resection	√	125	C	126	A	0.72 (0.47,1.10)	0.60 (0.41,0.86)
Chinot	2014	France	GB	Newly	Biopsy/Resection	√	458	D	463	B	0.88 (0.76,1.02)	0.64 (0.55,0.74)
Tham	2013	Australia	AO/AOA	-	Biopsy/Resection	√	36	B	26	A	1.03 (0.50,2.11)	1.29 (0.71,2.33)
Stupp	2014	Switzerland	GB	Newly	Biopsy/Resection	√	272	F	273	B	1.02 (0.81,1.29)	0.93 (0.76,1.13)
Kim	2011	Korea	GB	Newly	Biopsy/Resection	√	40	G	42	B	0.52 (0.24,1.12)	0.89 (0.49,1.62)
Hildebrand	2008	Belgium	AO/AOA	Newly	Biopsy/Resection	√	94	H	99	A	0.77 (0.56,1.06)	0.75 (0.57,0.99)
Levin	2000	USA	GBM	-	Biopsy/Resection	√	134	I	138	C	1.00 (0.90,1.10)	1.00 (0.90,1.10)
Muni	2010	Italy	GB	Newly	Biopsy/Resection	√	22	B	23	A	0.50 (0.26,0.99)	0.52 (0.29,0.93)
Kocher	2008	Germany	GB	-	Resection	√	29	B	33	A	0.84 (0.44,1.62)	0.91 (0.49,1.67)
Nabors	2015	USA	GB	Newly	Biopsy/Resection	√	88	F	89	B	0.69 (0.48,0.97)	0.82 (0.60,1.13)
Combs	2008	Germany	AO/AOA	-	Biopsy/Resection	√	20	B	40	A	1.51 (0.76,3.02)	1.86 (0.97,3.57)

HGG: High Grade Glioma (include GBM+AO/AOA); GB: Glioblastoma; AO/AOA: Anaplastic Oligodendroglioma/Oligoastrocytomas; LGG: Low Grade Glioma; GBM: Glioblastoma Multiforme; HR: Hazard Ratio; OS, Overall Survival; PFS, Progression-free Survival; -, none reported.

* A: Placebo; B: TMZ; C: PCV; D: BEV+TMZ; E: Nimotuzumab; F: Cilengitide+TMZ; G: TMZ+ACNU+CDDP; H: DBD+BCNU; I: DMFO+PCV; TMZ: Temozolomide; PCV: Procarbazine, Lomustine and Vincristine; BEV: Bevacizumab; ACNU: Nimustine; CDDP: Cisplatin; DBD: Dibromodulcitol; BCNU: Carmustine; DMFO: Eflornithine;

### Direct comparison

The results of pair-wise comparisons were illustrated in Table 2. There appeared to be no significant difference in OS status between patients treated with placebo and those treated by TMZ, PCV, Nimotuzumab or DBD in conjunction with BCNU (HR = 0.78, 95%CI = 0.56-1.11; HR = 0.81, 95%CI = 0.65-1.02; HR = 0.68, 95%CI = 0.42-1.11; HR = 0.77, 95%CI = 0.56-1.06). Also, there was no significant difference in OS status between patients treated with TMZ and those treated with BEV + TMZ, Cilengitide + TMZ or ACNU + CDDP +TMZ (HR = 0.88, 95%CI = 0.59-1.26; HR = 0.86, 95%CI = 0.58-1.26; HR = 0.52, 95%CI = 0.24-1.12). Similarly, introducing DMFO into PCV did not improve the effectiveness of PCV significantly (HR = 1.00, 95%CI = 0.91-1.11).

**Table 2:** Pair-wise meta-analyses of direct comparisons between the eight drugs

Endpoints	Direct comparisons	*I*^2^	Tau^2^	*P*_H_ values	HR (95% CI)	*P*_H_ values
**OS**	TMZ *vs*. Placebo	51.8%	0.076	0.081	0.78 (0.56, 1.11)	0.168
	PCV *vs*. Placebo	0.00%	0.000	0.517	0.81 (0.65, 1.02)	0.070
	Nimotuzumab *vs*. Placebo	-	-	-	0.68 (0.42, 1.11)	0.120
	DBD+BCNU *vs*. Placebo	-	-	-	0.77 (0.56, 1.06)	0.108
	BEV+TMZ *vs*. TMZ	-	-	-	0.88 (0.76, 1.02)	0.089
	Cilengitide+TMZ *vs*. TMZ	70.90%	0.056	0.064	0.86 (0.58, 1.26)	0.427
	TMZ+ACNU+CDDP *vs*. TMZ	-	-	-	0.52 (0.24, 1.12)	0.096
	DMFO+PCV *vs*. PCV	-	-	-	1.00 (0.91, 1.11)	1.000
**PFS**	TMZ *vs*. Placebo	80.90%	0.242	0.000	0.87 (0.53, 1.43)	0.581
	PCV *vs*. Placebo	0.00%	0.000	0.582	0.65 (0.53, 0.80)	0.000
	Nimotuzumab *vs*. Placebo	-	-	-	0.75 (0.49, 1.15)	0.191
	DBD+BCNU *vs*. Placebo	-	-	-	0.75 (0.57, 0.99)	0.041
	BEV+TMZ *vs*. TMZ	-	-	-	0.64 (0.55, 0.74)	0.000
	Cilengitide+TMZ *vs*. TMZ	0.00%	0.000	0.523	0.90 (0.76, 1.06)	0.217
	TMZ+ACNU+CDDP *vs*. TMZ	-	-	-	0.89 (0.49, 1.62)	0.702
	DMFO+PCV *vs*. PCV	-	-	-	1.00 (0.91, 1.11)	1.000

*H: heterogeneity; HR: hazard ratio; CI: confidence interval; OS: overall survive; PFS: progression-free survival; TMZ: Temozolomide; PCV: Procarbazine, Lomustine, and Vincristine; DBD: Dibromodulcitol; BCNU: Carmustine; BEV: Bevacizumab; ACNU: Nimustine; CDDP: Cisplatin; DMFO: Eflornithine;

Comparisons of PFS among different interventions were also listed in Table 2. PCV seemed to be the only intervention that significantly improved the PFS of glioma patients in comparison to the placebo (HR = 0.65, 95%CI = 0.53-0.80). Apart from this, introducing BEV into TMZ improved the PFS of glioma patients dramatically by 36% (HR = 0.64, 95%CI = 0.55-0.74). However, introducing other interventions into TMZ did not have considerable influence on the PFS of glioma patients (Cilengitide +TMZ *vs*. TMZ: HR = 0.90, 95%CI = 0.76-1.06; TMZ+ACNU+CDDP *vs*. TMZ: HR = 0.89, 95%CI = 0.49-1.62; DMFO+PCV *vs*. PCV: HR = 1.00, 95%CI = 0.91-1.11)

### Network meta-analysis

**Figure 3:**
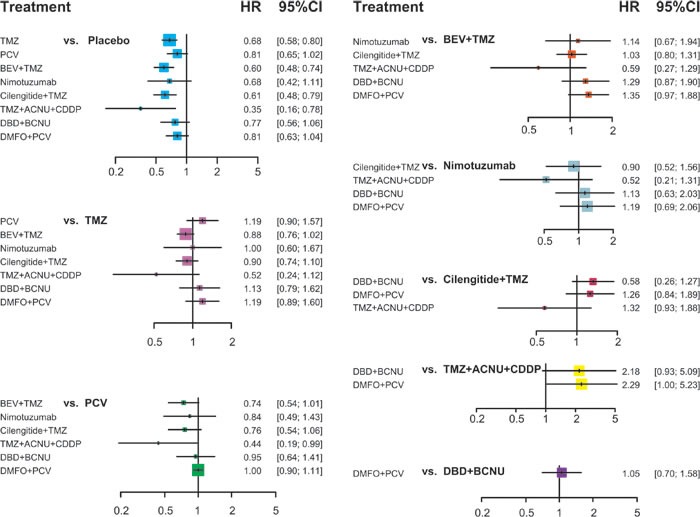
Plot of the HR of OS for different treatment strategies from the network meta-analysis.

**Figure 4:**
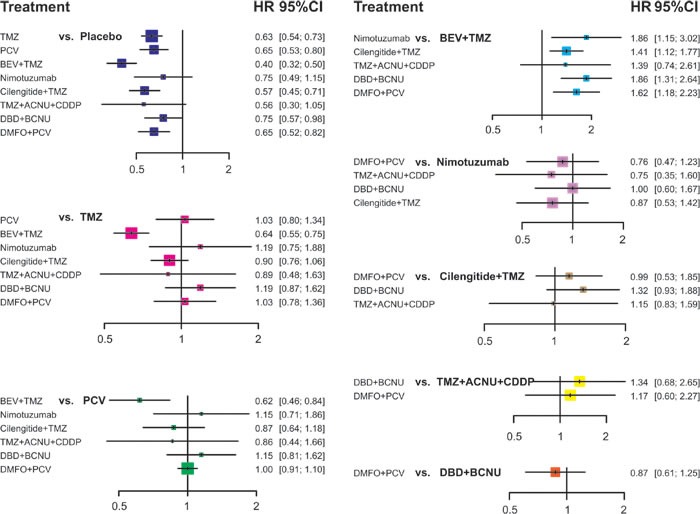
Plot of the HR of PFS for different treatment strategies from the network meta-analysis.

All mixed comparisons that synthesise both direct and indirect evidence are demonstrated in Table 3, Figure 3 and Figure 4. Placebo was proved to be significantly less effective than TMZ; BEV + TMZ; cilengitide + TMZ; TMZ, ACNU and CDDP. The corresponding HR and 95% *CrI* for these comparisons were 1.47 with 95%*CrI* 1.25 to 1.73, 1.67 with 95%*CrI* 1.34 to 2.08, 1.63 with 95%*CrI* 1.27 to 2.10 and 2.83 with 95%*CrI* 1.29 to 6.21. Additionally, PCV showed less effective compared to the combined usage of TMZ, ACNU and CDDP: HR values amounted to 2.29 with 95%*CrI* from 1.01 to 5.20. Apart from Nimotuzumab and TMZ + ACNU + CDDP, all other interventions were more effective than the placebo with respect to PFS of glioma patients. The corresponding HR values were listed as follows: TMZ with HR of 0.63 and 95%*CrI* of 0.54 to 0.73, PCV with HR of 0.65 and 95%*CrI* of0.53 to 0.80, BEV + TMZ with HR of 0.40 and 95%*CrI* of 0.32 to 0.50, cilengitide + TMZ with HR 0.57 and 95%*CrI* of 0.45 to 0.71, DBD + BCNU with HR of 0.75 and 95%*CrI* of 0.57 to 0.98 and DMFO + PCV with HR of 0.65 and 95%*CrI* of 0.52 to 0.82. Also, introducing BEV into TMZ significantly improved the PFS of glioma patients (HR = 0.64, 95%*CrI* = 0.55-0.75) and PCV remarkably improved the PFS of glioma patients (HR = 0.62, 95%*CrI* = 0.46 to 0.84). By contrast, nimotuzumab, cilengitide + TMZ, DBD + BCNU and DMFO + PCV appeared to be less effective than BEV + TMZ and the corresponding HR for the above comparisons were: 1.86, 95%*CrI* = 1.15-3.02; 1.41, 95%*CrI* = 1.12-1.77; 1.86, 95%*CrI* = 1.31-2.64; and 1.62, 95%*CrI* = 1.18-2.23.

**Table 3:** The efficacy (overall survival and progression-free survival) of eight drugs in chemotherapy of glioblastoma followed by surgery and radiotherapy according to the network meta-analysis using hazard ratio (HR) and corresponding 95% credible intervals (CrIs).

Endpoints	OS								
**PFS**	Placebo	**1.47 (1.25, 1.73)**	1.23 (0.98, 1.55)	**1.67 (1.34, 2.08)**	1.47 (0.90, 2.39)	**1.63 (1.27, 2.10)**	**2.83 (1.29, 6.21)**	1.30 (0.94, 1.79)	1.23 (0.96, 1.58)
	**0.63 (0.54, 0.73)**	TMZ	0.84 (0.64, 1.11)	1.14 (0.98, 1.32)	1.00 (0.60, 1.67)	1.11 (0.91, 1.35)	1.92 (0.89, 4.15)	0.88 (0.62, 1.26)	0.84 (0.62, 1.13)
	**0.65 (0.53, 0.80)**	1.03 (0.80, 1.34)	PCV	1.35 (0.99, 1.86)	1.19 (0.70, 2.04)	1.32 (0.94, 1.85)	2**.29 (1.01, 5.20)**	1.05 (0.71, 1.56)	1.00 (0.90, 1.11)
	**0.40 (0.32, 0.50)**	**0.64 (0.55, 0.75)**	**0.62 (0.46, 0.84)**	BEV+TMZ	0.88 (0.52, 1.50)	0.97 (0.76, 1.24)	1.69 (0.77, 3.71)	0.78 (0.53, 1.14)	0.74 (0.53, 1.03)
	0.75 (0.49, 1.15)	1.19 (0.75, 1.88)	1.15 (0.71, 1.86)	**1.86 (1.15, 3.02)**	Nimotuzumab	1.11 (0.64, 1.92)	1.92 (0.76, 4.85)	0.88 (0.49, 1.58)	0.84 (0.49, 1.45)
	**0.57 (0.45, 0.71)**	0.90 (0.76, 1.06)	0.87 (0.64, 1.18)	**1.41 (1.12, 1.77)**	0.76 (0.47, 1.23)	Cilengitide+TMZ	1.74 (0.78, 3.84)	0.80 (0.53, 1.20)	0.76 (0.53, 1.08)
	0.56 (0.30, 1.05)	0.89 (0.48, 1.63)	0.86 (0.44, 1.66)	1.39 (0.74, 2.61)	0.75 (0.35, 1.60)	0.99 (0.53, 1.85)	TMZ+ACNU+CDDP	0.46 (0.20, 1.07)	0.44 (0.19, 1.00)
	**0.75 (0.57, 0.98)**	1.19 (0.87, 1.62)	1.15 (0.81, 1.62)	**1.86 (1.31, 2.64)**	1.00 (0.60, 1.67)	1.32 (0.93, 1.88)	1.34 (0.68, 2.65)	DBD+BCNU	0.95 (0.63, 1.42)
	**0.65 (0.52, 0.82)**	1.03 (0.78, 1.36)	1.00 (0.91, 1.10)	**1.62 (1.18, 2.23)**	0.87 (0.53, 1.42)	1.15 (0.83, 1.59)	1.17 (0.60, 2.27)	0.87 (0.61, 1.25)	DMFO+PCV

**Figure 5:**
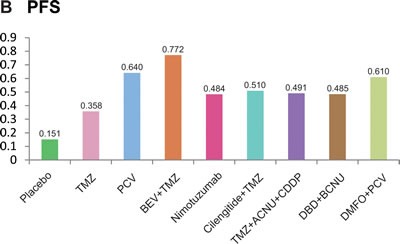
The cumulative ranking probabilities of different treatment strategies of OS and PFS.

**Figure 6:**
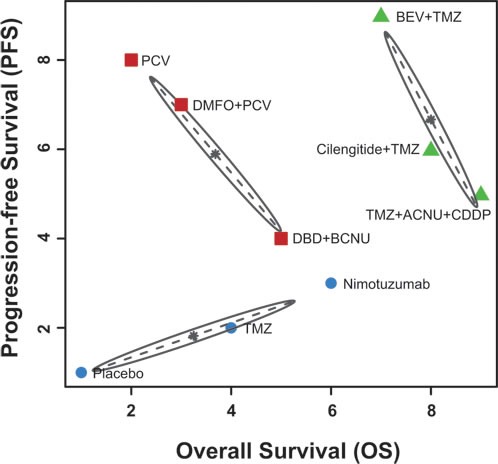
The cluster analysis of different treatment strategies of OS and PFS.

The estimated ranking probabilities of each intervention calculated through SUCRA were illustrated in Figure 5. As suggested by the corresponding SUCRA, the intervention of TMZ + ACNU + CDDP was ranked as the most effective treatment combination with respect to OS whereas the intervention of BEV + TMZ was ranked as the most effective one with respect to PFS. As a result, we performed a cluster analysis in order to simultaneously assess OS and PFS (Figure 6) and the corresponding treatments were categorized into three distinct clusters. The green cluster including BEV + TMZ, cilengitide + TMZ and TMZ + ACNU + CDDP appeared to have the most desirable OS and PFS whereas the blue cluster including nimotuzumab, TMZ and placebo seemed to be the least effective ones.

## DISCUSSION

In this multiple treatment Bayesian meta-analysis, eight chemotherapy interventions in combination with RT after surgery were tested in this research. A total of 3,681 patients suffering from glioma were involved in this research and these patients were obtained from 14 randomized control trials or clinical trials. Also direct together with indirect evidence were taken into consideration in order to complete the conventional meta-analysis. The aim of this analysis was to examine the optimal chemotherapeutic treatment for glioma and to provide confidential guidance for clinical practice through assessing and comparing the prognosis of patients.

As suggested by the rank probabilities of SUCRA, the combination of TMZ, ACNU and CDDP exhibited the most desirable OS. Another study conducted by Kim *et al.* [20] has identified the superiority of introducing both ACNU and CDDP into TMZ compared with single TMZ. Although TMZ, ACNU and CDDP were classified as alkylating agents, they had different mechanisms to control tumour cells. For instance, TMZ was converted into 5-(3-methyl)-1-triazen-1-yl-imidazole-4-carboximide (MTIC) through hydrolysis which provides affinity for guanine residues and induces either alkylation or methylation that further triggers apoptosis [33]. By contrast, ACNU is considered as the second-line chemotherapeutic intervention for glioma patients since it has comparable permeability for overcoming the blood-brain barriers. However, ACNU functions through the formation of DNA monoalkylated adducts which may lead to spontaneous depuration, single-strand breaks or alkali-labile sites [34]. Although ACNU was prone to guanine residues, it is able to inhibit DNA synthesis by cross-links [35]. However, damage caused by both TMZ and ACNU, especially the methylated product at O-6 position O^6^-methylguanine (O^6^MeG), can be restored by the DNA repair protein O^6^-methylguanine-DNA methyltransferase (MGMT) [36-38]. The presence of the MGMT enzyme prevented the apoptosis of tumour cells and suppressed the progression of both TMZ and ACNU [39]. On the other hand, CDDP can form multiple DNA adducts [40]. Unfortunately, this type of intervention is associated with several adverse effects. For instance, long term or high dosage of CDDP may induce cisplatin-resistant disease [41, 42] and it is acknowledged that CDDP has serious toxic effects such as ototoxicity and nephrotoxicity on patients [43]. Therefore, the combination of TMZ, ACNU and CDDP not only reduced their side effects on patients but also improved the prognosis of patients.

Unlike ACNU and CDDP, BEV is a humanized monoclonal antibody and it is the first angiogenesis inhibitor approved by the Food and Drug Administration (FDA). BEV is an antibody to VEGF-A, a predominant member of vascular endothelial growth factor (VEGF) family, which is highly expressed in tumour cells under hypoxia and could stimulate endothelial cell proliferation [44, 45]. Micro vessels regression, vessel growth and neovascularization inhibition can be achieved by anti-VEGF treatment. Since vascular proliferation was one of the pathological hallmarks of GBM, continuous BEV is important in glioma treatment due to its inhibition of angiogenesis [46]. Nevertheless, single BEV in clinical practices has not been confirmed by researches due to its poor penetration through blood-brain barriers. As suggested by some hypothesises, BEV may cross the blood-brain barrier and reach its target if other drugs with strong permeability such as TMZ were introduced and this may explain the excellent performance of BEV combined with TMZ [47].

This Bayesian network meta-analysis evaluates eight popular chemotherapy interventions that were incorporated into RT for managing glioma patients in clinical practices. The OS and PFS of glioma patients were compared through synthesizing both direct and indirect evidence in order to overcome issues such as small sample size and lack of head-to-head comparisons. Nevertheless, some limitations should not be neglected due to the nature of network meta-analysis. For instance, some chemotherapeutic interventions were intentionally excluded so that a closed loop of interventions can be formed for network meta-analysis. Besides that, five out of eight interventions-BEV + TMZ, nimotuzumab, TMZ + ACNU + CDDP, DBD + BCNU, DMFO + PCV-ontained only one eligible study and such a trend could have significant influence on the overall conclusions. Furthermore, factors such as different strategies of RT and the extent of surgery in each study were completely ignored and it is likely that the overall effectiveness of these chemotherapeutic may vary with the above mentioned confounding factors. Finally, our study did not take the modality of glioma into account and such an issue should be investigated by future researchers.

This study provided exclusive evidence that some chemotherapeutic agents including ACNU, CDDP or BEV can be introduced into TMZ in order to enhance its efficacy and hence improve the survival status of glioma patients. However, clinicians should be attentive to the characteristics of patients as well as the contraindications of chemotherapeutic interventions when selecting the most appropriate one for glioma patients.

## MATERIALS AND METHODS

### Search strategy

Medical databases including PubMed, Embase and Cochrane Library were consulted to identify all eligible randomized control trials that were not restricted by particular languages. The following searching terms with their corresponding synonyms were used to find relevant literatures: “glioma”, “radiotherapeutic treatment”, “surgery”, “chemotherapeutic agents”, “clinical trial” and “randomized control trial”. The reference list of every article was examined manually in order to prevent relevant articles being excluded and two reviewers (Ling Qi and Lijuan Ding) retrieved all the potential literature independently.

### Inclusion and exclusion criteria

Studies were eligible if they satisfied the following criteria: (i) they were randomized control trials, or clinical trials in phase II or III with more than 30 subjects; (ii) all the subjects were adults who were diagnosed with glioma, irrespective of different glioma types or grades; (iii) all chemotherapy drugs which were studied must be used with RT after surgery, although there was no restriction on the type of RT or the scope of surgery; (iv) studies must investigate the OS and PFS of glioma patients treated with at least two chemotherapeutic treatments or placebos. Additionally, even though the criteria above were met, studies were eliminated if they satisfied any exclusion criteria: (i) examined only one chemotherapy drug for which different delivery methods were compared such as intravenous and intra-arterial administration [13]; (ii) the reported interventions were not within a closed loop since indirect comparisons between two interventions cannot be established by linking them with a third intervention. The title and abstract of all the retrieved literatures were screened, and full text was also examined for the sake of determining the eligibility of studies. Two reviewers (Ling Qi and Lijuan Ding) performed all the above procedures independently and disagreement was resolved by discussion.

### Outcome measures and data extraction

For each eligible study, the following information was extracted: the main characteristics of the study including author; year of publication; country; type of study; sample size; baseline characteristics of patients such as glioma modality, newly or recurrent glioma, extent of surgery, type of RT, intervention details such as delivery methods and doses. Apart from these, interventions were evaluated using outcomes including median OS time, HR of OS, median time of PFS, HR of PFS as well as the number of adverse events occurred in each group.

Network meta-analysis (NMA) has been designed to compare the prognosis of glioma patients treated by different chemotherapeutic agents and primary endpoints incorporated in NMA were the OS and PFS for which the median value as well as the HR were compared. As defined by the National Cancer Institute (NIH), the OS time is the time between treatment commencements and patient death. OS is considered as a standard primary endpoint which is easy and precise to assess the overall effectiveness of interventions in oncologic clinical trials [14]. On the other hand, PFS is defined between the time at which disease was not progressed and the time of patient death. Both OS and PFS provide comprehensive evidence for the effectiveness of a specific chemotherapy. For those studies in which data of OS or PFS were not presented, we estimated these figures either using the cumulative survival percentages or the available survival curves.

### Statistical analysis

We set the significance level at α = 0.05 for all statistical tests. Heterogeneity between studies were assessed by the *Cochran's Q* statistic which calculates the weighted sum of squared differences [15] as:

$$Q=\sum \omega_{i}{\left(\theta_{i}-\theta \right)}^{2}$$

The proportion of the observed variance contributed by real differences in effect sizes rather than sampling errors was measured by the statistic of *I^2^* [16]:

$$I^{2}=\frac{Q-\left(k-1\right)}{Q}\times 100\%,\text{ where }k=\text{the number of studies}$$

In this case, the random-effects model was more appropriate than the fixed-effect model [17]. Subsequently, a NMA was conducted for both OS and PFS based on a Bayesian framework using R 3.2.3 software. Both direct and indirect evidence were synthesized in the NMA and the results were described as ORs with their corresponding 95% credible intervals. Furthermore, the surface under the cumulative ranking curve (SUCRA) was adopted to identify the optimal interventions with respect to different endpoints. The probabilities that each intervention ranks the best, second best and third best *etc*., were calculated in percentages and then these numerical values were accumulated in order to obtain their corresponding SUCRA values. All the investigated treatments were ranked based on their corresponding SUCRA values and a higher value of SUCRA provided evidence for more desirable OS and PFS time [18]. In addition, a cluster analysis was performed to group similar interventions by combining different endpoints.

## SUPPLEMENTARY MATERIALS TABLE


