# Supplementary material for: A network meta-analysis: the overall and progression-free survival of glioma patients treated by different chemotherapeutic interventions combined with radiation therapy (RT)
Source: Oncotarget. 2016 Jul 21;7(42):69002–13. doi: 10.18632/oncotarget.10763 (PMC5356607; doi:10.18632/oncotarget.10763)
Supplement: Supplementary file 1 [file oncotarget-07-69002-s001.docx]

A network meta-analysis: the overall and progression-free survival of glioma patients treated by different chemotherapeutic interventions combined with radiation therapy (RT)

**Supplementary Material**

| **Table S1. Jadad scale of 14 included studies.** | | | | | |
| --- | --- | --- | --- | --- | --- |
| **Author** | **Year** | **Country** | **Randomized** | **Blinded** | **Withdrawal** |
| Solomon | 2013 | Cuba | 2 | 2 | 0 |
| Stupp | 2005 | Switzerland | 2 | 0 | 0 |
| Van Den Bent | 2006 | Netherland | 2 | 0 | 1 |
| Shaw | 2012 | USA | 2 | 2 | 1 |
| Chinot | 2014 | France | 2 | 2 | 1 |
| Tham | 2013 | Australia | 0 | 0 | 0 |
| Stupp | 2014 | Switzerland | 2 | 0 | 1 |
| Kim | 2011 | Korea | 2 | 0 | 1 |
| Hildebrand | 2008 | Belgium | 2 | 0 | 1 |
| Levin | 2000 | USA | 2 | 0 | 0 |
| Muni | 2010 | Italy | 2 | 0 | 1 |
| Kocher | 2008 | Germany | 2 | 0 | 1 |
| Nabors | 2015 | USA | 2 | 0 | 1 |
| Comba | 2008 | Germany | 0 | 0 | 0 |
